# Nodular pulmonary deposition disease in a patient with the acquired immunodeficiency syndrome: a case report

**DOI:** 10.1186/s13256-020-02394-w

**Published:** 2020-06-04

**Authors:** Jessica N. Mezzanotte, I-Sanna Gibbons-Fideler, Konstantin Shilo, Mark Lustberg, Srinivas Devarakonda

**Affiliations:** 1 Department of Internal Medicine, The Ohio State University Wexner Medical Center, Columbus, OH USA; 2 Department of Pathology, The Ohio State University Wexner Medical Center, Columbus, OH USA; 3 Department of Infectious Disease, The Ohio State University Wexner Medical Center, Columbus, OH USA; 4 Department of Hematology, The Ohio State University Wexner Medical Center, Columbus, OH USA

**Keywords:** Light chain deposition disease (LCDD), AIDS, HIV, Hypergammaglobulinemia

## Abstract

**Background:**

Pulmonary nodules are a common cause for concern in patients with human immunodeficiency virus and acquired immunodeficiency syndrome. Most commonly, they are the result of an infection, given the patients’ immunocompromised state; however, in some cases, pulmonary nodules in patients with human immunodeficiency virus and patients with acquired immunodeficiency syndrome can result from cellular or protein deposits. We report a rare case of nodular pulmonary light chain deposition disease in a patient with acquired immunodeficiency syndrome and monoclonal gammopathy of undetermined significance.

**Case presentation:**

A 53-year-old African American woman with acquired immunodeficiency syndrome had pulmonary nodules detected incidentally by imaging of her lungs. Pulmonary tuberculosis was high on the differential diagnosis, but she had a negative test result for pulmonary tuberculosis. Imaging also revealed multiple lucent bone lesions, and earlier in the year, serum protein electrophoresis had shown an immunoglobulin G-kappa monoclonal protein (M spike). She was mildly anemic, so there was concern for progression to myeloma; however, the result of her bone marrow biopsy was unremarkable. Lung biopsy revealed finely granular eosinophilic material with negative Congo red staining, consistent with light chain deposition disease.

**Conclusions:**

The extent of this patient’s light chain deposition disease was thought to be caused by a combination of acquired immunodeficiency syndrome and monoclonal gammopathy of undetermined significance, and the interval decrease in lung nodule size after restarting antiretroviral therapy confirms this hypothesis and also highlights a potentially unique contribution of the hypergammaglobulinemia to this disease process in patients with human immunodeficiency virus and patients with acquired immunodeficiency syndrome .

## Background

Patients with plasma cell dyscrasias are capable of producing large amounts of free light chains [1]. In patients without an excessive production of free light chains, most excess light chains are rapidly cleared from the serum by glomerular filtration, which results in either their reabsorption and destruction by tubular cells or their excretion in the urine [2]. Occasionally, with plasma cell dyscrasias, the extremely large amount of free light chains produced can overwhelm the glomerular system, resulting in renal tubular damage and even overt renal failure [1]. Light chain deposition disease (LCDD) itself is most often a systemic disorder resulting from an underlying plasma cell or B-cell neoplasm [3]. LCDD is also often a diagnosis of exclusion, because amyloidosis must be ruled out by examination of fibrils for Congo red staining and apple green birefringence [3]. LCDD affects men more commonly than women and most often presents in systemic cases with renal manifestations [3]. Most extrarenal cases of systemic LCDD involve the heart, liver, and peripheral nervous system [3]. Localized LCDD is rare. Most cases of isolated, or localized, LCDD involve the kidney and skin, and cases of isolated pulmonary LCDD are rare, with less than 50 reported cases available in the literature, which is impressive because this phenomenon was first described in 1988 [4–6].

Of these rare cases of pulmonary LCDD, two separate histological patterns are appreciated—diffuse and nodular—with patients with nodular deposits having a better overall prognosis [5, 7]. Nodular pulmonary LCDD often appears similar to nodular amyloidosis radiographically and clinically, with the presentation involving either solitary or multiple pulmonary nodules in an otherwise asymptomatic patient [3]. These patients also often lack interstitial lung involvement that can be seen more commonly in systemic cases [3]. Additionally, nodular pulmonary LCDD has been shown to be more frequently associated with an underlying plasma cell dyscrasia or renal failure than pulmonary amyloidosis, with about 50% of cases of nodular pulmonary LCDD associated with an underlying plasma cell disorder, a low-grade B-cell lymphoproliferative disorder, or, in rare cases, even Sjögren syndrome [3, 5, 8]. Because our patient had monoclonal gammopathy of undetermined significance (MGUS), her diagnosis of nodular pulmonary LCDD fits within this small number of previously described cases.

## Case presentation

A 53-year-old African American woman with a past medical history significant for immunoglobulin G (IgG)-kappa MGUS, human immunodeficiency virus (HIV) infection progressive to acquired immunodeficiency syndrome (AIDS), and recent cerebrovascular accident with residual right-sided weakness presented to our hospital for evaluation of pulmonary nodules detected incidentally by imaging of her lungs. Of note, she had been receiving dolutegravir 50 mg twice-daily treatment for HIV but had not received abacavir-lamivudine for 5 months prior to presentation. Her dual CD4/CD3 count at the time of presentation was 148/mm^3^.

**Fig. 1:**
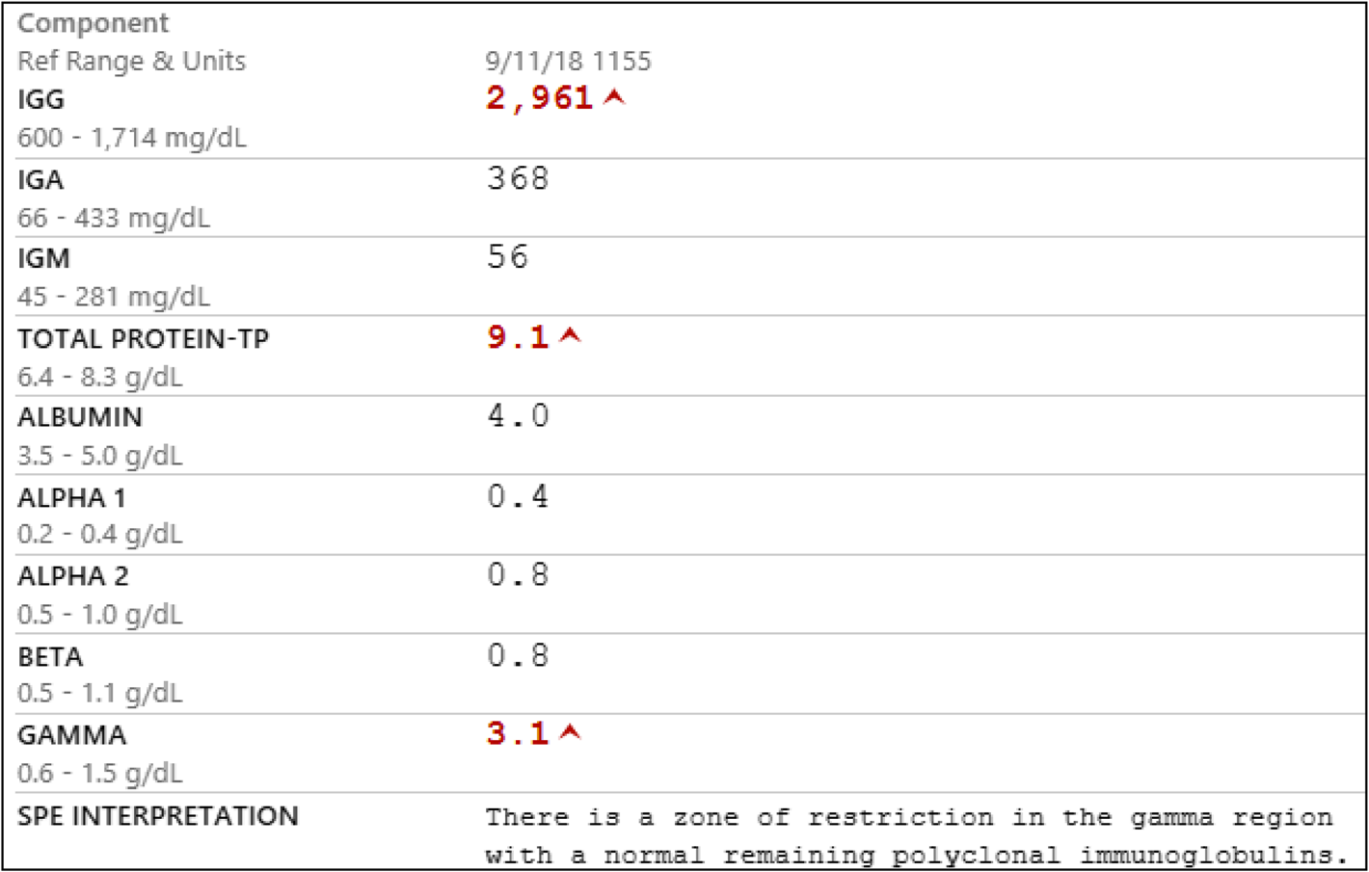
Results of monoclonal serum protein immunofixation. Results showed an elevated serum immunoglobulin G (IgG) level with the presence of an IgG-kappa monoclonal protein with a total serum monoclonal protein level of 327.6 mg/dl

Initially, concern for infection was high on the differential diagnosis, especially pulmonary tuberculosis (TB). She underwent an extensive infectious workup that included TB testing and later an autoimmune workup, the results of all of which were negative. Imaging studies also revealed multiple lucent bone lesions and osteopenia. She was found to be anemic at the time of evaluation, so there was concern that she had progressed to myeloma, as well. Serum monoclonal protein was elevated at 327.6 mg/dl; her serum protein electrophoresis is detailed in Fig. 1. Serum free light chain measurements were also elevated at 73.1 mg/L for both serum kappa and serum lambda free light chains with a serum kappa/lambda ratio of 1.0. Urine protein immunofixation was unable to be performed. Additional differential diagnoses at the time included lung cancer, plasmacytoma, and sarcoma.

**Fig. 2:**
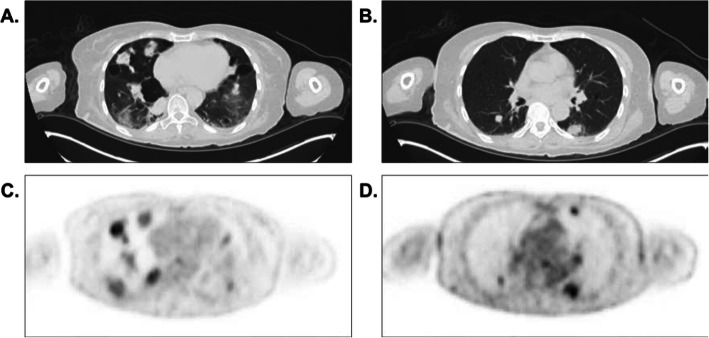
**a** and **b**. Computed tomography images with their corresponding positron emission tomography (PET) images (**c** and **d**) showing numerous pulmonary masses and nodules in the lower lung fields (**a** and **c**) and in the posterior segment of the left lower lobe (**b** and **d**)

**Fig. 3:**
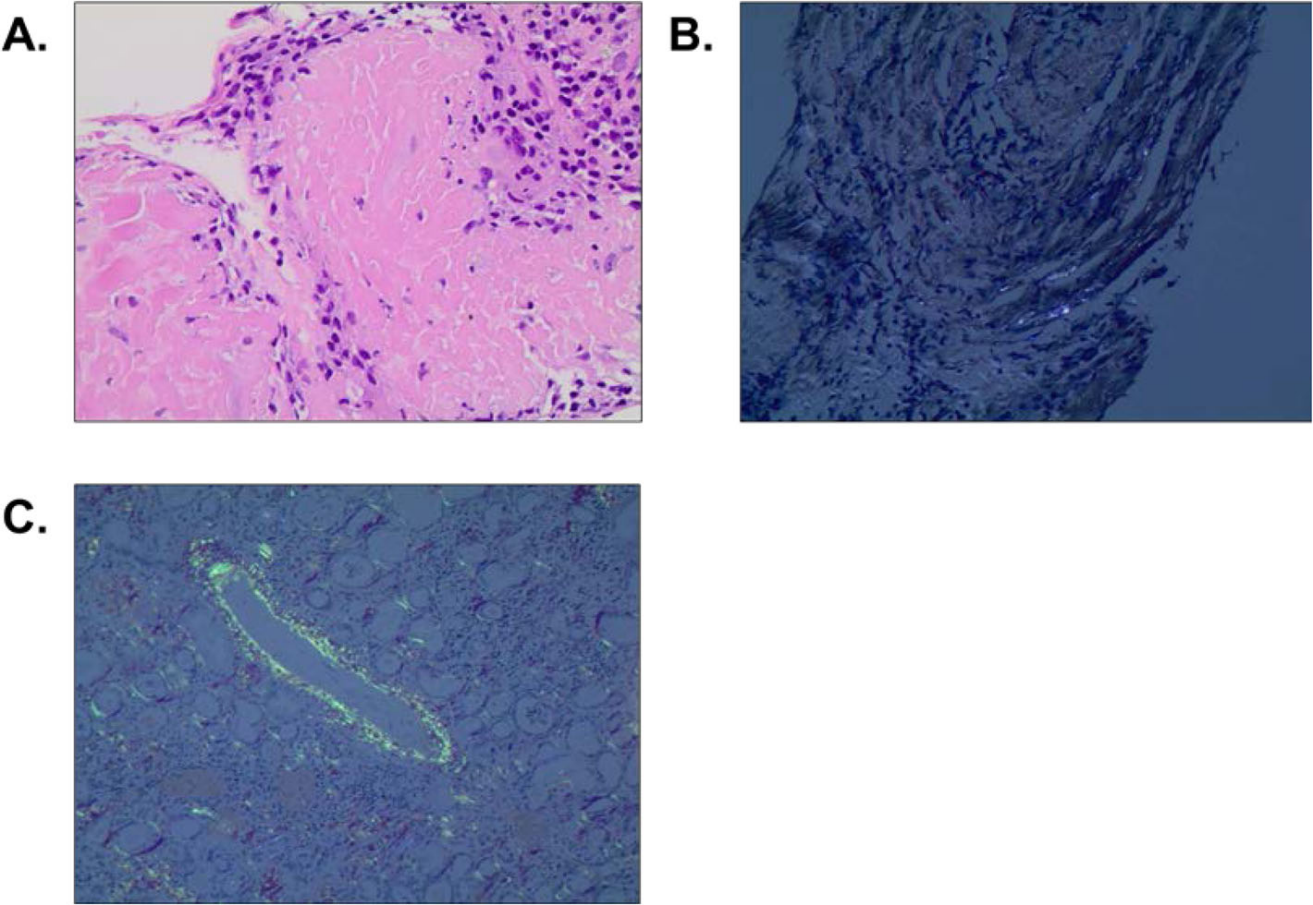
**a** and **b**. Computed tomography images with their corresponding positron emission tomography (PET) images (C and D) showing numerous pulmonary masses and nodules in the lower lung fields (**a** and **c**) and in the posterior segment of the left lower lobe (**b** and **d**)

To further examine the etiology of her lung nodules, she underwent positron emission tomography (PET), which confirmed multiple pulmonary masses and nodules with various degrees of fluorodeoxyglucose activity (Fig. 2). No definite focal hypermetabolic osseous lesion or lymphadenopathy was noted. She then underwent biopsies of both bone marrow and the lung nodules to further delineate the pathology of the findings. Bone marrow biopsy revealed 40% normocellular marrow with preserved trilineage hematopoiesis and mildly increased (5–10%) plasma cells that were polyclonal, not consistent with myeloma. Lung biopsy showed finely granular eosinophilic material consistent with LCDD (Fig. 3a). Staining was negative for Congo red (Fig. 3b, c); thus, pulmonary LCDD was the favored diagnosis over amyloidosis. The patient was discharged on dolutegravir 50 mg twice daily and efavirenz-emtricitabine-tenofovir 600-200-300 mg daily for antiretroviral therapy (ART). At her 6-month follow-up appointment, she reported good medication compliance, and computed tomography (CT) of her chest showed an internal decrease in size of all measurable pulmonary nodules.

## Discussion and conclusions

Nodular pulmonary deposits are not unique in patients infected with HIV, although the vast majority of pulmonary complications of patients with HIV and patients with AIDS are infectious in etiology [9, 10]. This case is unique, however, in that the patient had a negative test result for infection and had LCDD without any signs of amyloid features on biopsy. In addition to the patient’s MGUS as a contributing factor to her nodular pulmonary LCDD, her HIV infection progressive to AIDS could also have played a role by contributing to polyclonal B-cell proliferation, which is common in patients with HIV infection and contributes to hypergammaglobulinemia and lymphoid hyperplasia [6, 11]. This can produce plasma cell clones capable of producing nonamyloid immunoglobulin light chain deposits [6]. Indeed, recent studies show that HIV-positive patients have a significantly higher prevalence of monoclonal gammopathy than HIV negative individuals [11–13]. In fact, over 50% of patients treated with ART showed a decrease to total disappearance of serum monoclonal protein after 5 years of ART, and those with persistent monoclonal gammopathy were associated with higher levels of detectable plasma HIV RNA [12]. The fact that this patient’s HIV infection had been uncontrolled off abacavir-lamivudine and had progressed to AIDS could have contributed to the excessive levels of IgG-kappa in her serum, increasing the risk of developing systemic LCDD. It is therefore even more surprising that her only manifestation of the disease was asymptomatic pulmonary nodules.

This report therefore highlights a rare case of isolated nodular pulmonary LCDD in a patient with both MGUS and AIDS. Persistent HIV infection and high HIV viral load have been associated with increased B-cell dysfunction and hypergammaglobulinemia, which probably explains the persistence of nodular pulmonary LCDD seen off ART over the course of 3 months [12, 14, 15]. It is unclear why our patient’s nodules decreased in size after starting empiric treatment for clinical TB initially. There have been variable studies showing the effects of corticosteroids and cytotoxic medications on pulmonary LCDD [16], but we suspect that the interval decrease in size of the nodules was actually due to the reinitiation of ART with the combination of efavirenz, emtricitabine, and tenofovir (Atripla; Gilead Sciences, Foster City, CA, USA) and dolutegravir, because this has previously been reported in the literature [17, 18]. Overall, this case highlights the rare combination of B-cell dysfunction and hypergammaglobulinemia associated with uncontrolled HIV infection that can lead to systemic manifestations such as the nodular pulmonary deposits seen in this patient.

## Data Availability

Not applicable.

## Abbreviations

AIDS: Acquired immunodeficiency syndrome
ART: Antiretroviral therapy
CT: Computed tomography
HIV: Human immunodeficiency virus
IgG: Immunoglobulin G
LCDD: Light chain deposition disease
MGUS: Monoclonal gammopathy of undetermined significance
PET: Positron emission tomography
TB: Tuberculosis
